# Rational Drug Prescribing a Way to Reduce Patients Out of Pocket Payment

**Published:** 2018-03

**Authors:** Ali SOROUSH, Mohammad MOHSENI, Majeed NAKHAEE, Farideh MORADI

**Affiliations:** 1. Life Style Modification Research Center, Imam Reza Hospital, Kermanshah University of Medical Sciences, Kermanshah, Iran; 2. Health Management and Economics Research Center, Iran University of Medical Sciences, Tehran, Iran; 3. Dept. of Health Services Management, School of Health Management and Information Sciences, Iran University of Medical Sciences, Tehran, Iran

## Dear Editor-in-Chief

Health is known as one of the main preconditions for the social welfare system, people to achieve the level of health in the incidence of sickness, in addition, to keep appropriate lifestyle, use health services and on the other way, they try to buy those services. Taking advantage of these services impose different financial pressures on people in different societies, these fees paid directly out of pocket sometimes may cause financial problems for patients and their families (1).

Direct paying out of pocket is the simplest and least effective methods of payment, in this way the patient pays the money to the service provider at the time of service delivery (2). Paying a high-cost out-of-pocket refrains people from purchasing essentials such as food and clothing and every year almost 44 million households and more than 150 million people face with high cost of health expenses and almost 25 million households and more than 10 million people are taken into extreme poverty (3).

One of the methods leading to patients over-charging is lack of physicians’ attention to logical prescription (4). Unnecessary prescribing drugs include prescribing many different drugs, prescribing drugs without indication, prescribing lower or more than the drug’s dose, prescribing expensive drugs, vitamins and antibiotics, unnecessary prescription of injectable forms of drug, administration drug during treatment with more or less than the prescribed limit, prescribed generic drugs in terms of efficacy, prescribing drugs with intervention and weakness and problem in how to take the drugs (5). Irrational drug use can threaten the health of people’s personal and social, in addition to making financial losses. About 1.5% to 43.5% of inpatients and 2% to 50% of outpatients have drug adverse side effects. The drug adverse side effects can lead to longer hospitalization time increasing patients’ out of pocket pay (6).

One of the most common models having many fans for reforming the health system is using the levers of control. Control levers describe separate areas of the structure and functioning of the health system, which is very important regarding the performance, and governments can manipulate them, these levers cover different topics such as financing, payment systems, organizing and regulation of rules and behaviors (7). One of the common methods of fee payment at hospitals is using the fee for service system, in this form of payment of each unit and clinical procedures such as injections, laboratory tests, and so on. This method gives an incentive payment to do more service-to-service providers (7).

In order to decrease patients’ out of pocket payment being created because of unnecessary hospital stay, it is recommended using a combination of pay for performance and fee for services can be used. Therefore, an expert supervisory team is composed in hospital and assessing the performance of physicians regarding daily visits and timely discharge and while providing appropriate feedback to any of the doctors and declaring physicians performed well in this regard to all doctors and a percent of the payment to them will be allocated to evaluation of performance score.
